# How Does Internet Use Improve Mental Health among Middle-Aged and Elderly People in Rural Areas in China? A Quasi-Natural Experiment Based on the China Health and Retirement Longitudinal Study (CHARLS)

**DOI:** 10.3390/ijerph192013332

**Published:** 2022-10-16

**Authors:** Shishuai Fan, Yifan Yang

**Affiliations:** 1 School of Public Affairs, Zhejiang University, Hangzhou 310058, China; 2 School of Public Administration, Southwest Jiaotong University, Chengdu 610031, China

**Keywords:** middle-aged and elderly people, Internet use, mental health, difference-in-differences

## Abstract

One of the most significant public health issues in rural China is how to improve the mental health of middle-aged and older individuals. Using 2013, 2015, and 2018 CHARLS panel data, this paper properly examined the effects of Internet use on the mental health of middle-aged and elderly people in rural China based on the difference-in-differences method. The findings are as follows: (1) Internet use effectively improves the mental health status of middle-aged and elderly people in rural China; (2) compared to the middle-aged group, Internet use has a more obvious effect on the mental health of the elderly; (3) further analysis showed that reading news, watching videos, and playing games online could significantly improve the mental health status of middle-aged and elderly people in rural China, while chatting online and other Internet activities cannot significantly improve mental health status; and (4) playing games, watching videos, and reading news have different effects on the mental health of middle-aged and elderly people in rural China. The results indicate that playing games have a better effect on depression levels than watching videos. In contrast, watching news had the lowest effect on depression levels among middle-aged and elderly people in rural China. The results of this study also show the latest evidence that Internet use can help China’s rural middle-aged and elderly populations to reduce social isolation, establish new social connections, gain social support, and, ultimately, achieve active ageing. Therefore, promoting multiple forms of interaction is an effective path to prevent loneliness, which has become the new policy direction of the government to create an age-friendly Internet environment using various measures in the future to eliminate the barriers to Internet access affecting the middle-aged and elderly in rural China.

## 1. Introduction

Health is the basis for active ageing among the elderly. According to the definition of health created by the World Health Organization (WHO), “health is a state of complete physical, mental and social well-being and not merely the absence of disease or infirmity” [1]. As an essential and integral part of health, mental health is one of the important focuses of public health. During economic and social transition periods, urbanization, and mass migration, the polarization of income and wealth as well as low levels of social security have contributed to widespread anxiety and poor mental health, especially for the elderly in rural China. The findings of the Annual Report on Elderly Health in China show that more than 30% of the elderly have a high risk of depression, and the elderly in rural areas have a higher risk of depression [2]. The poor mental health status of the rural elderly is also reflected in the suicide rate of the rural elderly group, which is much higher than urban and other age groups [3].

There are many factors affecting the mental health of the elderly in rural China. The departure of young people from rural areas during the modernization process is often considered a key factor contributing to the poor mental health status of the elderly in rural China [4]. Different from Western society, support from one’s children is one of the important pillars for the survival of the elderly in rural China under the long-term influence of traditional filial culture [5]. As large numbers of young people leave the countryside for cities, it is harder for the rural elderly to receive support from their children, which inevitably affects the traditional security system, and the mental health of the rural elderly is also affected. A great deal of previous research into how to improve mental health among the elderly in rural China has focused on support from one’s children. For example, Shen and Zhang argue that grandchildren’s caregiving frequency was negatively associated with depressive symptoms [6]; Xu suggests that living with children is beneficial to the mental health of the rural elderly [7]. He maintains that financial support from one’s children and emotional support had a significant positive impact on the mental health of the elderly [8]. Yoh indicates that intergenerational programs, such as the company of children and financial support children, could serve as key health promoters among elderly people by decreasing the risk of social isolation and loneliness due to a greater sense of meaningfulness [9].

However, these studies still regard financial support from one’s children as well as their company and ability to provide care as necessary ways to improve the mental health of the elderly in rural China [10], which is not achievable, and the decline of support from one’s children is a mainstream trend. Therefore, some scholars have begun to seek other ways to improve the mental health of the elderly in rural China. Better social services [11,12], social security [13,14], and environments that are conducive to healthy ageing [15,16,17] are considered to be important factors in improving the mental health of the elderly [18]. The proliferation of smartphones and the Internet has also provided a new opportunity to improve the mental health of elderly people. The China Statistical Report on Internet Development indicates that the Internet coverage rate in China has increased rapidly in the past five years. Specifically, the Internet popularity rate in China was only 53.2% in 2016, but it increased rapidly to 73% in 2021. The proportion of Internet users aged 60 and above has also increased rapidly, from 4% in 2016 to 11.5% in 2021 [19,20]. Additionally, several studies have begun to examine the effects of Internet use on the mental health of elderly people. 

Older people can improve their mental health by finding entertainment and making friends through the Internet. Some previous studies have shown that surfing the Internet can effectively improve the mental health status of the elderly in rural China. Li points out that rural adults can find a large amount of health information, increase their social interaction, and maintain physical exercise to improve their health by using the Internet [21]; using national data from the China Family Panel Study in 2016 and the propensity score-matching approach, Wang suggests that older adults who reported that used the Internet have lower depression levels than those who do not use the Internet and that it is critical to advocate for technology-based policies and programs that promote older adults’ Internet use to improve their social well-being [22]. Lyu studied 7193 older adults aged 60–95 years old and found that Internet use was positively associated with the self-assessed health of older adults, and social capital was an important mediating variable [23]. However, other studies suggest that Internet use not only does not improve mental health in older adults, but even worsens it because increased time spent online erodes elderly people’s social interactions and leads to a reduction in social participation, a narrowing of social circles, and a weakening of the sense of community belonging and networks of friends. Xie’s study found that Internet use increased the risk of depression in older adults, and further heterogeneity analysis reveals that the negative effects on mental health are more evident for specific groups of older adults, such as those who are women, younger and middle-aged, high-income, non-rural Hukou, less educated, and living with others [24]. Baker and Algorta emphasize that some Internet behaviors such as negative interactions and social comparisons may exacerbate the rebellion of some Internet users against mainstream social values, promote loneliness and alienation, and cause indifference and fear [25].

The exploration of these studies strongly supports the continuation of this study, but there are also some shortcomings. This study argues that the different research samples and the limitations of research methods have contributed to the inconsistency of these studies: First of all, cross-sectional data are mostly used for OLS regression analysis to examine the relationship between Internet use and the mental health status of the elderly. However, in addition to Internet use of the elderly, other conditions, such as the overall improvement of the social environment, will also improve the mental health status of the elderly, which cannot solve many endogeneity problems. Secondly, some studies have tried to use panel data to investigate the impact of Internet access on the mental health of the elderly. However, due to the limited number of samples and the limitations of the database itself, the results that have been obtained are not convincing [26]. Fortunately, CHARLS data provide a better natural experiment when combined with the multiphase DID method, which can solve the problem of an insufficient sample size of elderly participants and eliminate endogenous problems caused by missing variables. There can be more scientific and standardized exams on the impact of Internet use on China’s rural elderly mental health. In addition, the mental health of some rural middle-aged people who are about to enter the elderly demographic is also worthy of attention, so this paper further expands the scope of the research sample. We try to answer whether Internet access can improve the mental health status of rural middle-aged and elderly people. Furthermore, this study examines the effects of different online activities, such as chatting, playing games, and watching the news, on the mental health of the rural elderly.

## 2. Materials and Methods

### 2.1. Samples and Data Sources

This study used panel data from the China Health and Retirement Longitudinal Study (CHARLS) collected in 2013, 2015, and 2018. CHARLS is a nationally representative database of Chinese residents aged 45 and older sponsored by the National Development Research Institute of Peking University and co-organized by the China Social Science Research Center of Peking University and is the most popular database for studying the health of middle-aged and elderly people in China. The baseline data in CHARLS were collected in 2011 and include about 10,000 households and 17,500 individuals from 150 counties/districts and 450 villages/resident committees [27]. The 2013 sample covered 10,606 households and 18,311 respondents, of which 6347 households and 10,995 respondents were from rural areas. The 2015 sample covered 12,058 households and 20,860 respondents, of which 7187 households and 12,452 respondents were from rural areas. The 2018 sample covered 11,510 households and 19,670 respondents, of which 6866 households and 11,749 respondents were from rural areas [28]. The sample is restricted to exclude respondents with key missing values for “Internet use” and “mental health” covered in the model, and respondents who had previously participated in the survey were also excluded. The final estimating sample consists of 29,909 observations from 237 villages in rural eastern, central, and western China collected between 2013 and 2018.

### 2.2. Model Construction

**Figure 1:**
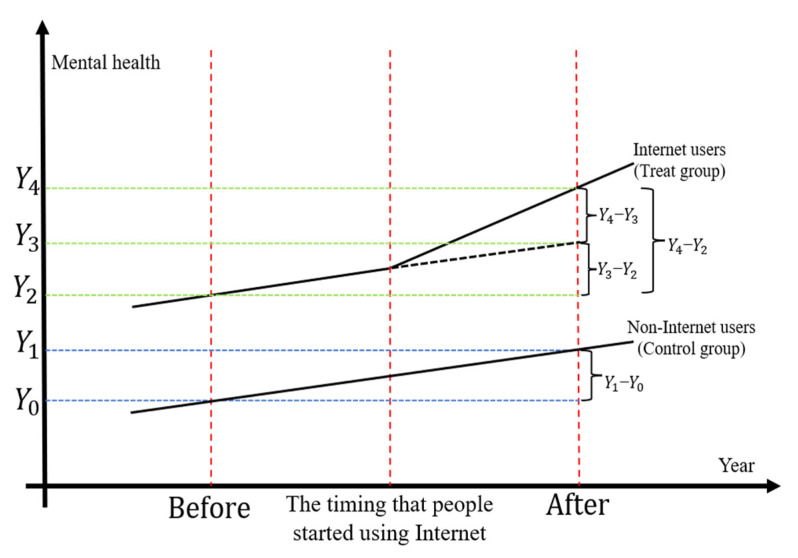
The rationale for the DID method.

The most direct way to examine the impact of Internet use on the mental health of middle-aged and elderly people in rural China is to compare mental health levels before and after Internet use. However, this approach is also affected by endogenous issues such as economic development and environmental change. To eliminate the interference of various endogenous factors, this paper refers to Beck’s research and adopts the difference-in-differences (DID) method [29]. The key of the DID method is to find a control group, treatment group, and a corresponding intervention measure. In this paper, we identified middle-aged and elderly people who used the Internet as the treatment group and those who did not use the Internet as the control group. Internet use is the intervention. Therefore, the mental health levels of the middle-aged and elderly people in the control group were not affected by Internet use. Figure 1 illustrates the rationale for the DID method. In Figure 1, the net effect of Internet use on improving the mental health of middle-aged and elderly people in rural China is *Y*_4_ − *Y*_3_. *Y*_3_ − *Y*_2_ is the improvement in mental health caused by endogenous factors such as improved living standards and environment that do not belong to the intervention effect of Internet use. In reality, however, we can only observe the effect of *Y*_4_ − *Y*_2_ after Internet use, and the effect of endogenous factors such as environmental improvement is wrongly included, so the obtained effect of Internet intervention is biased. According to the DID method, the improving effect of unobservable endogenous factors such as environmental improvement can be replaced by the control group, that is, (*Y*_3_ − *Y*_2_) = (*Y*_1_ − *Y*_0_). Therefore, the net effect of Internet use is obtained through (*Y*_4_ − *Y*_2_) − (*Y*_1_ − *Y*_0_).

The empirical model for the DID method is expressed as:

(1) $$Mental\;health_{it}=\alpha_{0}+\beta_{1}DID_{it}+\beta_{2}Z_{it}^{\prime }+\gamma_{i}+\lambda_{t}+\varepsilon_{it}$$

where $Mental\;health_{it}$ denotes the mental health of the No.i observation at time t; $\alpha_{0}$ is a constant term; $DID_{it}$ is the independent variable, and specifically, $DID_{it}$ = $Treat_{i}$ × $Time_{t}$, $Treat_{i}$ is a dummy variable that takes a value of 1 if observation i belongs to the treatment group; $Time_{t}$ is also a dummy variable of time before or after Internet use; therefore, $\beta_{1}$ indicates the pure effect of Internet use on mental health. To ensure the effectiveness of the DID results, we control several factors that are likely to affect the mental health of middle-aged and elderly people in rural China, as represented by $Z_{it}^{\prime }$. $\gamma_{i}$, which are individual fixed effects that simply control for omitted variables that vary among individuals, but not across time; $\lambda_{t}$ are time-fixed effects and allow bias to be eliminated from the unobservable variables that change over time but that are constant over entities, and it controls for factors that differ across entities but that are constant over time [30]; $\varepsilon_{it}$ is the error term.

### 2.3. Variables and Operation

#### 2.3.1. Internet Use

The 2013, 2015, and 2018 surveys all asked respondents “have you used the Internet in the last month?”. Respondents answered “yes” or “no”, with “yes” being assigned a value of 1, and “no” being assigned a value of 0. In this study, respondents who answered “yes” were set as the treatment group, and those who answered “No” were set as the control group. In the 2018 survey, the respondents were further asked “What do you usually do on the Internet?” There are five options in the questionnaire, including chatting, watching the news, and watching videos, which provide the possibility to further explore how different Internet activities affect the mental health of middle-aged and elderly people in rural China.

#### 2.3.2. Mental Health

Mental health is a dependent variable. The international and universal “Center for Epidemiological Survey, Depression Scale” (CES-D) was used to measure the mental health levels in the CHARLS data. There are 10 questions in the CES-D that reflect the mental health of the elderly. Two questions reflect a positive mental health status: “I felt hopeful about the future” and “I was happy”. Additionally, the remaining eight are negative indicators: “I was bothered by things that do not usually bother me”, “I had trouble keeping my mind on what I was doing”, “I felt depressed”, and so on. Each question has four answers: “rarely or none of the time”, “some or a little of the time”, “occasionally or a moderate amount of the time”, and “most or all of the time”, which are represented by the values of 0~3, and the two positive questions were assigned in the reverse direction. The values of the 10 questions were summed to reflect the mental health status. The higher the value, the worse the mental health status of the elderly and the more serious the depression tendency.

#### 2.3.3. Control Variables

According to previous studies on the factors that influence mental health [31,32], this paper selected control variables from two aspects: individual characteristics and family characteristics. The variables for individual characteristic include age, gender, marital status, education level, frequency of contact with children, taking care of grandchildren, pension, and chronic diseases. The variables of family characteristics include family economic level and family size. All the control variables are defined in Table 1.

## 3. Results

### 3.1. Descriptive Analysis

The summary statistics are listed in Table 2 and cover the number of observations, means, and standard deviations across 2013, 2015, and 2018. Table 2 also lists the means of the variables divided between the treatment group for Internet use and the control group. Firstly, the mental health level of the treatment group is significantly better than that of the control group. Secondly, the age of the treatment group was significantly lower than that of the control group, and the prevalence of chronic diseases was also lower in the treatment group than in the control group. In addition, the education level and consumption level of the treatment group were significantly higher than they were in the control group. However, there was little difference between the treatment group and the control groups regarding marital status, frequency of contact with children, care for grandchildren, pension, and family size.

### 3.2. DID Estimation

As shown in Table 3, column (1) is the average treatment effect (ATE) result estimated by the panel DID model. Columns (1) and (2) of Table 3 control for both individual and time-fixed effects to eliminate some endogeneity issues that vary over time and across individuals. According to the results of column (1), the coefficient of the interaction term of the DID model was −1.304, with a significance value of 1%, which indicates that Internet use effectively improved the mental health level of middle-aged and elderly people in rural China. The goodness of fit (R^2^) of the DID model was 0.687, and the adjusted R^2^ was 0.502, indicating that the explanation level of the DID model is very good. The F value of the DID model is 10.83, which indicates that the DID model is very significant. Moreover, in column (2), the results are still robust after the addition of other control variables, and the coefficient of the interaction term of the DID model was −1.320, with a significance value of 1%. In terms of control variables, a high frequency of meetings or contact with children effectively improved the mental health status of middle-aged and elderly people in rural China. However, the coefficient of suffering from chronic disease is 0.636, with a significance value of 1%, which indicates that suffering from chronic diseases had a positive leading effect on the depression level of middle-aged and elderly people in rural China. The goodness of fit (R^2^) of the DID model after the control variables were added was 0.685, and the adjusted R^2^ was 0.503, which is very similar to column (1). The F value of the DID model is 5.024, which indicates that the DID model is very significant.

### 3.3. Heterogeneous Effect

Table 4 further demonstrates the impact of Internet use on the mental health levels of two different groups of middle-aged and elderly people in rural China. The results of column (1) show, where there are no control variables, that the mental health level of middle-aged adults in rural China is negatively associated with Internet use and is statistically significant at the 1% level. When control variables are covered in the regression equation, the regression coefficient value of (Treat × Post) will be up-regulated from −1.199 to −1.209, remaining robust and statistically significant at the 1% level (column (2)). The results of columns (3) and (4) show that Internet use has the same effect on the mental health of the elderly as that of the middle-aged. Firstly, the results of columns (3) show that where there are no control variables, the mental health level of elderly people in rural China is negatively associated with Internet use and statistically significant at the 5% level. Secondly, when control variables are covered in the regression equation, the regression coefficient value of (Treat × Post) will be up-regulated from −3.368 to −3.433, which remains robust and statistically significant at the 5% level (column (4)). In addition, comparing the results of column (2) and column (4), it is found that Internet use has a better effect on the mental health of the elderly than that of middle-aged people.

### 3.4. Further Analysis

The results of previous empirical studies show that Internet use has a significant effect on the mental health of middle-aged and elderly people in rural China and that the depression levels of middle-aged and elderly people in rural China have decreased significantly. However, many activities can be completed online, such as chatting, reading news, watching videos, and playing games, among others. What online activities are related to the improved mental health of middle-aged and elderly people in rural China? In other words, different online activities may lead to different mental health levels, which is why previous studies determining the effects of Internet use on the mental health of middle-aged and elderly people in rural China have produced different results. To test the impact of different online activities on mental health levels, this paper adopted the difference-in-difference-in-difference (DDD) method to conduct further empirical analysis [33]. The model is set as follows:

(2) $$Mental\;health_{it}=\alpha_{0}+\beta_{1}DID_{it}\;\times \;\mathrm{IA}+\beta_{2}Z_{it}^{\prime }+\gamma_{i}+\lambda_{t}+\varepsilon_{it}$$

The only difference between Formulas (1) and (2) is that the IA (Internet activity) option is added, which includes chatting, reading news, watching videos, playing games, and activities. Chat activities are indicated by the question “have you been chatting online in the past month?”. Respondents answered “yes” or “no”, with “yes” being assigned a value of 1 and “no” being assigned a value of 0. Using this method, online news consumption is indicated by the question “have you been reading news online in the past month?”. Respondents answered “yes” or “no”, with “yes” being assigned a value of 1 and “no” being assigned a value of 0. Whether the respondents watched videos online was indicated via the question “have you been watching videos online in the past month?”. Respondents answered “yes” or “no”, with “yes” being assigned a value of 1 and “no” being assigned a value of 0. Whether or not the respondents played games online or not was indicated with the question “have you been playing games online in the past month?”. Respondents answered “yes” or “no”, with “yes” being assigned a value of 1 and “no” being assigned a value of 0. Participation in other online activities was indicated with the question “have you been doing other activities online in the past month?”. Respondents answered “yes” or “no”, with “yes” being assigned a value of 1 and “no” being assigned a value of 0.

Table 5 shows the regression results of the impact of different online activities on the mental health of middle-aged and elderly people in rural China. In column (1), the coefficient of the interaction term Treat × Post × Chat) in the DDD model is −0.480, but it is not significant, indicating that chatting online cannot improve the mental health level of middle-aged and elderly people in rural China. In column (2), the coefficient of Treat × Post × News is −1.126, which is significant at the 10% level, indicating that reading news online can significantly improve the mental health of middle-aged and elderly people in rural China. In column (3), the coefficient of Treat × Post × Videos is −1.735, which is significant at the 5% level, indicating that watching videos online can significantly improve the mental health level of middle-aged and elderly people in rural China. In column (4), the coefficient of Treat × Post × Games is −2.852, which is significant at the 1% level, indicating that playing games online can significantly improve the mental health of middle-aged and elderly people in rural China. In column (5), the coefficient of Treat × Post × Others (0.204) is not significant, indicating that other forms of online activities do not affect the mental health level of middle-aged and elderly people in rural China. The goodness of fit (R^2^) of the DDD model was 0.684, and the adjusted R^2^ was 0.503, indicating that the explanation level of the DDD model is very good. The F values of this DDD model are close to 5, which indicates that the DID model is very significant.

## 4. Robustness Test

### 4.1. Pre-Tend Test

The key hypothesis identified from the panel DID is that the treatment group and the control group show the same development trend before the policy was implemented. In other words, if the development trend is different, it means that other factors may affect the changes in the dependent variables. To this end, this paper draws on existing methods to test the parallel trend between the treatment group and the control group [34,35]. The specific model is set as follows:

(3) $$Mental\;health_{it}=\sigma_{0}+\sum_{s=-2+}^{2+}\sigma_{s}DID_{s}+\beta_{2}Z_{it}^{\prime }+\gamma_{i}+\lambda_{t}+\varepsilon_{it}$$

**Figure 2:**
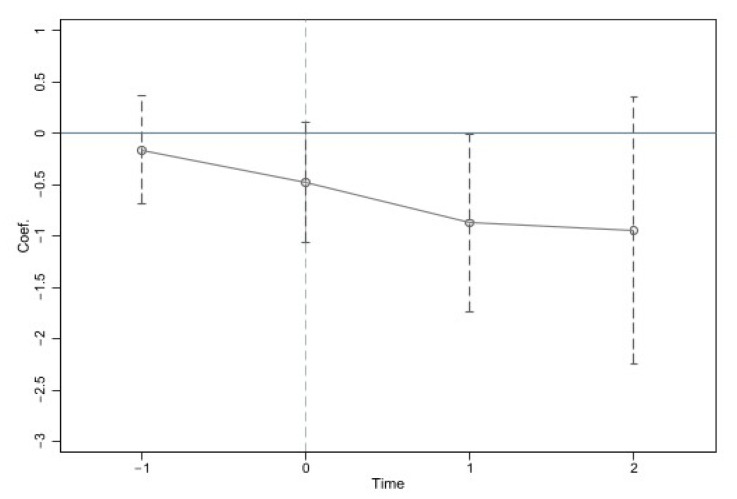
Parallel trend test results.

In Equation (3), $\sigma_{s}$ is the estimated coefficient of concern in this paper, which shows the difference in time trend between the treatment group and the control group; DID is a dummy variable; and S represents the time window when middle-aged and elderly people in rural China received Internet access. When S is positive, it represents the s year after receiving Internet access. When s is negative, it means the s year before Internet access. The value of DID is only 1 in year S and is 0 in other years. It is worth noting that *S* =−2 (the first two years during which middle-aged and elderly people are online) is set as the base year. Other variables are consistent with Formula (1). Figure 2 shows the estimation results of Equation (3). It can be found from Figure 2 that the estimated coefficient of the mental health level of middle-aged and elderly people in rural China is not significant in all periods before they go online. After Internet access, the alleviating effect of Internet access on the depression of middle-aged and elderly people in rural China gradually emerged and showed a downward trend year by year.

### 4.2. Placebo Effect Test

**Figure 3:**
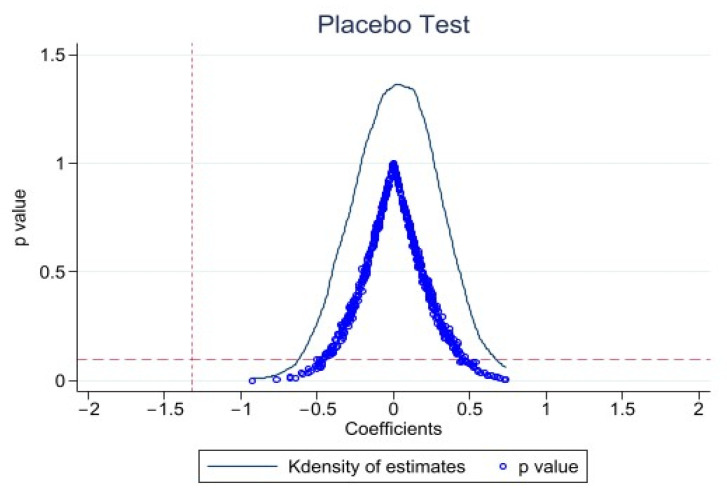
Placebo test.

Another possible cause of estimation bias is omitted variables. Referring to the research of Cai [36] and La Ferrara [37], this study randomly selected middle-aged and elderly people in rural China from the sample to conduct a placebo test on the main results of this paper. Accordingly, we randomly selected 842 individuals from 27,242 middle-aged and elderly individuals and set them as the “virtual” online observation, which was affected by Internet use, and set the remaining individuals as the observation not affected by Internet use. A dummy variable $Treat_{i}^{False}$ for the placebo test and a cross term $Treat_{i}$ × $Post_{t}$ for the placebo test were constructed accordingly. Since the “dummy” treatment group was randomly generated, the placebo test cross-term should not have a significant effect on the dependent variable, that is, βfalse = 0. If the estimated coefficient of βfalse deviates from zero statistically significantly, it indicates that there is identification bias in the model set. At the same time, to avoid the interference of other small probability events on the estimation results, we repeated the above process 500 times for regression analysis. Figure 3 shows the coefficients of kernel density of estimates and the distribution of corresponding *p*-values for the treatment group of 500 random generations. The mean value of the regression coefficient is close to 0 (coefficient is 0.017), and most *p* values are greater than 0.1. The actually estimated coefficients represented by the vertical bars in Figure 3 are outliers in the estimated coefficients of the placebo test, and the estimated results are not seriously biased by the omitted variables.

## 5. Discussion

Different from previous studies, this paper examines the effects of Internet use on the mental health of middle-aged and elderly people in rural China through a quasi-natural experiment. The study found that Internet use significantly improved mental health and reduced depression levels among middle-aged and elderly people in rural China, which is consistent with many previous studies [38]. Compared to the middle-aged group, Internet use has a better effect on the mental health of the elderly. The possible reason for this result is that the middle-aged group has a broader social network and can obtain more social support and social connections through employment and other means, while the elderly group has fewer ways to maintain social connections and to receive social support. Due to the decreasing intergenerational support in real life, the Internet has become a key channel of spiritual support for the elderly. Further research has shown that watching news, watching videos, and playing games can significantly improve the mental health of middle-aged and elderly people in rural China, while other online activities such as online chatting do not affect the mental health of middle-aged and elderly people in rural China, which adds to the gaps in previous research. This is because online chatting is still a way for acquaintances to socialize, and it is difficult for middle-aged and elderly people in rural China to make new friends online through chat software such as WeChat or QQ. Playing games, watching videos, and reading news have different effects on the mental health of middle-aged and elderly people in rural China. The results indicate that playing games has a better effect on depression levels than watching videos. In contrast, watching news had the lowest effect on depression levels among middle-aged and elderly people in rural China.

According to previous studies, more opportunities for social participation and social support are the main ways to improve the mental health levels of middle-aged and elderly people in rural China [39]. Therefore, the possible reasons for this result are that middle-aged and elderly people in rural China have different levels of social participation and social support in different online activities. Watching news can help middle-aged and elderly people in rural China have access to more external information but a lower level of social support. However, this kind of information transmission is one-way, and middle-aged and elderly people in rural China who are in a state of social isolation cannot build new social networks and increase social participation through reading news. Due to the rise of short video and live-streaming platforms such as Tiktok and Kwai, however, watching videos online can not only help middle-aged and elderly people in rural China to obtain outside information, but also to relax and entertain themselves by watching videos, which is more likely to generate temporary social interaction and increase social participation through bullet chatting. As such, watching videos improves mental health levels more than reading news does. Playing online games can effectively relieve depression in middle-aged and elderly people in rural China, with the best possible reason for this being that the process of playing online games results in more social interactions with unfamiliar online friends, setting up relatively long-term and stable social networks while also providing access to more social support and increasing social participation.

This paper also makes the following research contributions: Firstly, this paper proposes that Internet use is a new support method for the mental health of middle-aged and elderly people in China, which is an effective supplement to the previous intergenerational family support. This is a new trend for future development and enriches the existing research. Second, the research has several methodological and data advantages. Based on the CHARLS panel data from 2013, 2015, and 2018, this study adopted the DID method to study the impact of Internet use on the mental health of middle-aged and elderly people in rural China and determined parallel trends and placebo tests, effectively avoiding many endogeneity problems and making the research results more scientific and credible. Third, this paper extends the scope of the study to include middle-aged people who are about to enter old age in rural China. With the exception of age, the characteristics of the two groups are similar, making it easier to provide suggestions for future policy interventions. Fourth, Internet activities are diverse, but different Internet activities have different effects on mental health, which is one of the important reasons for the differences in previous research results. By using the DDD method, this paper studies the effects of different online activities, such as online chatting and online games, on the mental health of middle-aged and elderly people in rural China.

However, there are still some shortcomings in this study. Although this study uses CHARLS panel data for middle-aged and elderly people in China, Internet use is not fully popular among middle-aged and elderly people, and some samples are missing from the panel data, so the sample comprising the treatment group is still small, which may affect the final estimated coefficient. Secondly, Internet use is a long-term behavior, and this study only has three periods of panel data and does not study the long-term impact of Internet use on the mental health status of middle-aged and elderly people in rural China. Thirdly, Internet activities are very diverse. Limited by the questionnaire design of CHARLS, this paper can only distinguish Internet activities into five categories: online chatting, watching the news, watching videos, playing games, and other online activities, without the ability to make a more diverse and detailed distinction. Finally, the mechanism of the effect of Internet use on the mental health status of middle-aged and elderly people in rural China is not clear, that is, we are not sure why Internet use can improve the mental health status of middle-aged and elderly people in rural China. These shortcomings also point to future research directions.

## 6. Conclusions

The mental health of middle-aged and elderly people in rural China is one of the key public health problems in rural China. In the context of declining intergenerational support, it is important to improve the mental health status of middle-aged and elderly people in rural China through other methods. This paper uses CHARLS panel data from 2013, 2015, and 2018 to accurately investigate the impact of Internet use on the mental health of middle-aged and elderly people in rural China based on the DID method. The findings of this study are as follows: (1) Internet use effectively improves the mental health status of middle-aged and elderly people in rural China; (2) compared with the middle-aged group, Internet use has a more obvious effect on the mental health of the elderly; (3) further analysis showed that reading news, watching videos, and playing games online could significantly improve the mental health status of middle-aged and elderly people in rural China, while Internet chatting and other online activities did not significantly improve mental health status. (4) Playing games, watching videos, and reading news have different effects on the mental health of middle-aged and elderly people in rural China. The results indicate that playing games has a better effect on depression level than watching videos. In contrast, watching news had the lowest effect on depression levels among middle-aged and elderly people in rural China: 1.126. The findings add to existing research showing the latest evidence of the impact of Internet use on the mental health of middle-aged and elderly people in rural China. 

At the same time, the results of this study also provide new directions for the government, communities, NGOs, and families to improve the mental health of middle-aged and elderly people in rural China [40]. First of all, we need to accelerate the construction of an age-friendly environment in rural areas, especially the construction of communication infrastructure and digital libraries, and to improve the Internet access conditions of middle-aged and elderly people in rural China. The government also needs to encourage telecom operators to introduce more preferential telecommunications tariff policies for rural areas, reduce the consumption costs of Internet use for the elderly in rural areas, and encourage them to actively participate in Internet activities. Second, more Internet software should be updated and modified for ageing and provide more products with features such as large fonts, large icons, and high-contrast text for the middle-aged and elderly. More enterprises should be encouraged to introduce interface modes with simple interfaces that are easy to operate, enabling various barrier-free functions such as one-click operation and text input prompts to lower the technological threshold for middle-aged and elderly people to access the Internet. Last but not least, support from family members is still an important way to improve the mental health of middle-aged and elderly people in rural China. While establishing a new social network and providing social support from strangers, the Internet can also serve as a bridge between left-behind groups and family members who have left rural China. Therefore, the community, social organizations, and families should actively guide the elderly to overcome psychological barriers and to master Internet skills through various activities that can help middle-aged and elderly people integrate into Internet life. All of these measures will allow the Internet to become an effective tool to help middle-aged and elderly people in rural China to achieve active ageing.

## Figures and Tables

**Table 1:** Definition of control variables.

Variable Name	Variable Symbol	Definition
Age	Age	Unit: years
Gender	Gender	Female = 0, male = 1
Marry status	Marry	Married = 1, divorced = 0
Education level	Education	Illiterate = 0, primary = 6, middle school = 9, high school = 12, college = 16
Frequency of contact with children	Contact	Almost never = 0, once a year = 1, once every six months = 2, once every three months = 3, once a month = 4, every two weeks = 5, once a week = 6, 2–3 times a week = 7, almost every day = 8
Taking care of grandchildren	Care	Yes = 1, no = 0
Expenditure	Expenditure	Log (per capita consumption expenditure)
Pension	Pension	Yes = 1, no = 0
Suffering from chronic disease	Disease	Yes = 1, no = 0
Family size	Family	The number of people living together in a household

**Table 2:** Descriptive statistics of the variables.

Variable	Total			Control Group			Treat Group		
	N	Mean	SD	N	Mean	SD	N	Mean	SD
Age	29,909	61.09	9.522	28,903	60.98	9.433	841	52.43	5.798
Gender	29,909	0.485	0.500	29,067	0.478	0.500	842	0.730	0.444
Marry	29,909	0.809	0.393	29,067	0.807	0.395	842	0.879	0.326
Education	28,861	4.544	3.850	28,095	4.410	3.788	766	9.457	2.723
Contact	29,909	6.917	1.698	29,067	6.913	1.700	842	7.033	1.606
Care	29,909	0.415	0.493	29,067	0.415	0.493	842	0.400	0.490
Expenditure	29,572	8.777	1.236	28,738	8.761	1.236	834	9.344	1.121
Pension	29,909	0.829	0.377	29,067	0.828	0.377	842	0.850	0.357
Disease	29,909	0.706	0.456	29,067	0.709	0.454	842	0.586	0.493
Family	29,795	3.239	2.346	28,955	3.240	2.363	840	3.219	1.635
Mental health	29,909	8.715	6.386	29,067	8.793	6.398	842	6.010	5.300

**Table 3:** Baseline results of DID estimates.

	(1)	(2)
	Depression Level	Depression Level
Treat × Post	−1.304 ***	−1.320 ***
	(−3.29)	(−3.27)
Gender		−0.204
		(−0.23)
Age		0.014
		(0.66)
Marriage status		−0.254
		(−1.60)
Education level		−0.033
		(−1.00)
Contact with children		−0.054 *
		(−1.83)
Care grandchildren		0.039
		(0.41)
Expenditure		0.084 **
		(2.35)
Pension		−0.024
		(−0.23)
Chronic disease		0.636 ***
		(5.08)
Family size		−0.030
		(−1.24)
Constant	8.747 ***	7.638 ***
	(896.04)	(5.21)
Observations	29,909	27,242
Individual FE	Yes	Yes
Year FE	Yes	Yes
R^2^	0.687	0.685
Adj R^2^	0.502	0.503
F statistics	10.83	5.024

Notes: Standard errors are clustered by household and are listed in parentheses. *** *p* < 0.01, ** *p* < 0.05, * *p* < 0.1.

**Table 4:** DID estimates for different groups.

	(1)	(2)	(3)	(4)
	Middle-Aged Group		Elderly Group	
	Depression Level	Depression Level	Depression Level	Depression Level
Treat × Post	−1.199 ***	−1.209 ***	−3.368 **	−3.433 **
	(−2.93)	(−2.88)	(−2.12)	(−2.35)
Constant	8.406 ***	5.323 **	9.159 ***	9.159 ***
	(486.34)	(2.16)	(1740.90)	(4.60)
Observations	16,352	14,393	12,443	11,793
Control	No	Yes	No	Yes
Individual FE	Yes	Yes	Yes	Yes
Year FE	Yes	Yes	Yes	Yes
R^2^	0.692	0.688	0.674	0.675
Adj R^2^	0.508	0.509	0.484	0.486
F statistics	8.612	2.816	4.515	2.717

Notes: Standard errors are clustered by household and are listed in parentheses. *** *p* < 0.01, ** *p* < 0.05.

**Table 5:** DDD results of the impact mechanism analysis.

	(1)	(2)	(3)	(4)	(5)
	Depression Level	Depression Level	Depression Level	Depression Level	Depression Level
Treat × Post × Chat	−0.480				
	(−0.60)				
Treat × Post × News		−1.126 *			
		(−1.93)			
Treat × Post × Videos			−1.735 ***		
			(−3.01)		
Treat × Post × Games				−2.852 **	
				(−2.15)	
Treat × Post × Others					0.204
					(0.21)
Constant	8.516 ***	8.530 ***	8.532 ***	8.523 ***	8.508 ***
	(13.92)	(13.94)	(13.95)	(13.93)	(13.91)
Observations	27,427	27,427	27,427	27,427	27,427
Control	Yes	Yes	Yes	Yes	Yes
Individual FE	Yes	Yes	Yes	Yes	Yes
Year FE	Yes	Yes	Yes	Yes	Yes
R^2^	0.684	0.684	0.684	0.684	0.684
Adj R^2^	0.503	0.503	0.503	0.503	0.503
F statistics	4.449	4.809	5.347	4.897	4.412

Notes: Standard errors are clustered by household and are listed in parentheses. *** *p* < 0.01, ** *p* < 0.05, * *p* < 0.1.

## Data Availability

The data from the 2013, 2015, and 2018 China Health and Retirement Longitudinal Study (CHARLS) are publicly available at https://charls.charlsdata.com/pages/data/111/en.html, accessed on 29 June 2022.

## References

[1] WHO World Health Organization (WHO) Definition Of Health. https://www.publichealth.com.ng/world-health-organizationwho-definition-of-health/.

[2] Liu Y. (2021). Annual Report on Elderly Health in China.

[3] NHC China Health Statistics Yearbook 2021. https://data.cnki.net/yearbook/Single/N2022010155.

[4] Zuo D., Li S. (2011). The Impact of Labor Migration on Healthy Well-being of Elderly Left Behind in Rural China: Studies Based on Surveys in Inflow and Outflow Places. J. Public Manag..

[5] Fei X. (1983). The Problem of Elderly Supporting in the Change of Family Structure. J. Peking Univ. Philos. Soc. Sci..

[6] Shen L., Zhang Z. (2020). The Grandparenting and Mental Health of Middle Aged and Elderly People: The Mediating Effect of Family Cohesion. Stud. Psychol. Behav..

[7] Xu Q. (2018). Living Arrangement and Depression among the Chinese Elderly People: An Empirical Study Based on CHARLS. Sociol. Rev. China.

[8] He Q., Tan Y., Peng Z. (2021). How Does Grandchild Care Affect the Mental Health of Grandparents?—New Evidence from CHARLS. Popul. Dev..

[9] Murayama Y., Ohba H., Yasunaga M., Nonaka K., Takeuchi R., Nishi M., Sakuma N., Uchida H., Shinkai S., Fujiwara Y. (2015). The effect of intergenerational programs on the mental health of elderly adults. Aging Ment. Health.

[10] Shu Z., Xiao J., Dai X., Han Y., Liu Y. (2021). Effect of family” upward” intergenerational support on the health of rural elderly in China: Evidence from Chinese Longitudinal Healthy Longevity Survey. PLoS ONE.

[11] Lv X., Zhang X. (2022). The Influence of Community Home-based Elderly Care on the Health of the Elderly Population. Chin. J. Popul. Sci..

[12] Wang X., Liu M., Li Y., Guo C., Yeh C.H. (2020). Community canteen services for the rural elderly: Determining impacts on general mental health, nutritional status, satisfaction with life, and social capital. BMC Public Health.

[13] Li Y., Wang Z., Xiang Y. (2022). The Effect of the Social Health Insurance on the Mental Health of the Middle-Aged and Aged Residents in Rural China—An Empirical Analysis Based on CHARLS. Financ. Econ..

[14] Wang L., Liu C. (2022). Research on the Integration of Medical Insurance For urban and Rural Residents, Health and Health Inequality for the Rural Elderly. Soc. Secur. Stud..

[15] Lu S., Wang L. (2021). Research on the Effect of Urban and Rural Communities Environment on the Mental Health of the Elderly. Popul. Dev..

[16] Jiang W., Sun J. (2022). Living Style, Living Environment and Mental Health of Urban and Rural Elderly—An Analytical Framework for the Construction of an Elderly Friendly Community. Urban Probl..

[17] Zhang R., He X., Liu Y., Li M., Zhou C. (2022). The Relationship between Built Environment and Mental Health of Elderly People: The Mediating Effects of Perceptions of Community Cohesion and Community Safety and the Moderating Effect of Income. Front. Public Health.

[18] Shao M., Chen J., Ma C. (2022). Research on the Relationship between Chinese Elderly Health Status, Social Security, and Depression. Int. J. Environ. Res. Public Health.

[19] CNNIC The 39th Statistical Report on China’s Internet Development. http://www.cnnic.net.cn/hlwfzyj/hlwxzbg/hlwtjbg/201701/P020170123364672657408.pdf.

[20] CNNIC The 49th Statistical Report on China’s Internet Development. http://www.cnnic.net.cn/hlwfzyj/hlwxzbg/hlwtjbg/202202/P020220721404263787858.pdf.

[21] Li L., Zeng Y., Zhang Z., Fu C. (2020). The impact of internet use on health outcomes of rural adults: Evidence from China. Int. J. Environ. Res. Public Health.

[22] Wang Y., Zhang H., Feng T., Wang H. (2019). Does internet use affect levels of depression among older adults in China? A propensity score matching approach. BMC Public Health.

[23] Lyu S., Sun J. (2021). Internet use and self-rated health among Chinese older adults: The mediating role of social capital. Geriatr. Gerontol. Int..

[24] Xie L., Yang H.-L., Lin X.-Y., Ti S.-M., Wu Y.-Y., Zhang S., Zhang S.-Q., Zhou W.-L. (2021). Does the Internet Use Improve the Mental Health of Chinese Older Adults?. Front. Public Health.

[25] Baker D.A., Algorta G.P. (2016). The relationship between online social networking and depression: A systematic review of quantitative studies. Cyberpsychol. Behav. Soc. Netw..

[26] Zhang H., Wang H., Yan H., Wang X. (2021). Impact of Internet Use on Mental Health among Elderly Individuals: A Difference-in-Differences Study Based on 2016–2018 CFPS Data. Int. J. Environ. Res. Public Health.

[27] Zhao Y., Hu Y., Smith J.P., Strauss J., Yang G. (2014). Cohort profile: The China health and retirement longitudinal study (CHARLS). Int. J. Epidemiol..

[28] Zhao Y., Strauss J., Xinxin C., Yafeng W., Jinquan G., Qinqin M., Gewei W., Huali W. China Health and Retirement Longitudinal Study Wave 4 User’s Guide. https://charls.charlsdata.com/Public/ashelf/public/uploads/document/2018-charls-wave4/application/CHARLS_2018_Users_Guide.pdf.

[29] Beck T., Levine R., Levkov A. (2010). Big bad banks? The winners and losers from bank deregulation in the United States. J. Financ..

[30] Angrist J.D., Pischke J.-S. (2009). Mostly Harmless Econometrics: An Empiricist’s Companion.

[31] Yang Y., Deng H., Yang Q., Ding X., Mao D., Ma X., Xiao B., Zhong Z. (2020). Mental health and related influencing factors among rural elderly in 14 poverty state counties of Chongqing, Southwest China: A cross-sectional study. Environ. Health Prev. Med..

[32] Zhang C., Giles J., Zhao Y. (2015). Policy Evaluation of China’s New Rural Pension Program: Income, Poverty, Expenditure, Subjective Wellbeing and Labor Supply. China Econ. Q..

[33] Ma S., Guo J. (2022). How do Institutional Innovations Affect China’s Cross-border E-commerce Exports? Evidence from the Establishment of Cross-border E-commerce Comprehensive Pilot Area. J. Manag. World.

[34] Li P., Lu Y., Wang J. (2016). Does flattening government improve economic performance? Evidence from China. J. Dev. Econ..

[35] Liu Q., Qiu L.D. (2016). Intermediate input imports and innovations: Evidence from Chinese firms’ patent filings. J. Int. Econ..

[36] Cai X., Lu Y., Wu M., Yu L. (2016). Does environmental regulation drive away inbound foreign direct investment? Evidence from a quasi-natural experiment in China. J. Dev. Econ..

[37] La Ferrara E., Chong A., Duryea S. (2012). Soap operas and fertility: Evidence from Brazil. Am. Econ. J. Appl. Econ..

[38] Li L., Ding H., Li Z. (2022). Does Internet Use Impact the Health Status of Middle-Aged and Older Populations? Evidence from China Health and Retirement Longitudinal Study (CHARLS). Int. J. Environ. Res. Public Health.

[39] Zhao L., Wu L. (2022). The Association between Social Participation and Loneliness of the Chinese Older Adults over Time—The Mediating Effect of Social Support. Int. J. Environ. Res. Public Health.

[40] Pan J., Yang Y. (2021). Research on the Holistic Governance of the Elderly Digital Inclusive Society. J. Southwest Jiaotong Univ. Soc. Sci..

